# Natural History of the Slave Making Ant, *Polyergus lucidus*, *Sensu lato* in Northern Florida and Its Three *Formica pallidefulva* Group Hosts

**DOI:** 10.1673/031.007.4201

**Published:** 2007-07-17

**Authors:** Joshua R. King, James C. Trager

**Affiliations:** ^1^ Department of Biological Science, Florida State University, Tallahassee, FL 32306-4370; ^2^ Shaw Nature Reserve, PO Box 38, Interstate 44 and Hwy 100, Gray Summit, MO, 63039

**Keywords:** body size, colony size, conservation, sociometry, taxonomy, *Formica archboldi*, *Formica dolosa*

## Abstract

Slave making ants of the *Polyergus lucidus* Mayr (Hymenoptera: Formicidae) complex enslave 3 different *Formica* species, *Formica archboldi*, *F*. *dolosa*, and *F. pallidefalva*, in northern Florida. This is the first record of presumed *P*. *lucidus* subspecies co-occurring with and enslaving multiple Formica hosts in the southern end of their range. The behavior, colony sizes, body sizes, nest architecture, and other natural history observations of *Polyergus* colonies and their *Formica* hosts are reported. The taxonomic and conservation implications of these observations are discussed.

## Introduction

In North America, the formicine ant genus *Polyergus*, commonly referred to as Amazon ants, consists of 2 species (which include several so-called subspecies) of obligate social parasites of the genus *Formica*. Dulosis, also called slave-making, is the characteristic behavioral feature of *Polyergus* and involves workers periodically raiding *Formica* species' nests for brood, especially pupae. *Polyergus* species are obligate social parasites, wholly dependent on the host (enslaved) species to carry out all of the tasks necessary for colony function (foraging, maintenance, brood rearing). This form of social parasitism is unusual among ants but has evolved several times independently in the ant subfamilies Myrmicinae and Formicinae.

The eastern North American *Polyergus* species, *Polyergus lucidus* Mayr, (Hymen optera: Formicidae) may best be described as a species complex (Smith 1947; Trager et al. in press). The distribution of *P*. *lucidus* overlaps that of its hosts throughout their range. The *Formica* hosts for *P*. *lucidus* are all in the *pallidefulva* group (Trager et al. in press). This group includes 5 species [*F*. *archboldi*, *F*. *dolosa*, *F*. *incerta*, *F*. *pallidefulva*, *F*. sp. nov (described in Trager et al. in press)], all free-living. There are no records of colonies of *P*. *lucidus*, *s.l*. containing more than one slave species. Records for *P*. *lucidus* are spotty and there have been very few records of different hosts in close geographic proximity. One exception to this pattern is a population of *P*. *lucidus* on Long Island, New York with 3 hosts (*F*. *dolosa*, *F*. *incerta*, *F*. *pallidefulva*) that has been previously studied by Howard Topoff and his students (Kwait and Topoff 1984; Goodloe et al. 1987 using host names *schaufussi* for *dolosa* and *nitidiventris* for *incerta* and *pallidefiilva*).

In Florida, *P*. *lucidus* has been recorded in 4 counties and is generally considered a rare species throughout the southeastern U.S. (Deyrup 2003) and its entire range (Creighton 1950; JCT unpublished records). There are host records for *F*. *archboldi* (Trager and Johnson 1985), *F*. *dolosa*, and *F*. *pallidefulva* from different sites in the southern range limit of *P*. *lucidus*, but no records of *P*. *lucidus* enslaving multiple hosts at the same locality. Colony collections are particularly rare. The focus of much of the previous study of this and other *Polyergus* species has been on the raiding behavior of workers (Talbot 1968a; Marlin 1969; Kwait and Topoff 1984), mating and colony founding (Talbot 1968b; Marlin 1968, 1971; Topoff et al. 1988), and host specificity (Goodloe and Topoff 1987). Trager and Johnson (1985) reported on most of these topics for a Florida population of *Polyergus* that was hosted by *F*. *archboldi*. By comparison, there has been little study of the sociometry, natural history, and colony-level attributes of *Formica* host species and the 5 known *Polyergus* species worldwide, in spite of the popularity of these species as examples of the evolution of social parasitism in ants (Hölldobler and Wilson 1990, D'Ettorre and Heinze 2001).

Here, we report the first record of sympatry for *P*. *lucidus* on three host species in the southern part of its range. This includes a second record of *P*. *lucidus* enslaving *F*. *archboldi*, a species endemic to the southeastern U.S. (Creighton 1950; Trager and Johnson 1985). The relative abundance, natural history, in-nest behavior, and some basic sociometric data (colony sizes, worker sizes, nest architecture, queen egg laying rates) are described for 3 host *Formica* species and *P*. *lucidus* colonies in northern Florida.

## Materials and Methods

**Figure 1.**
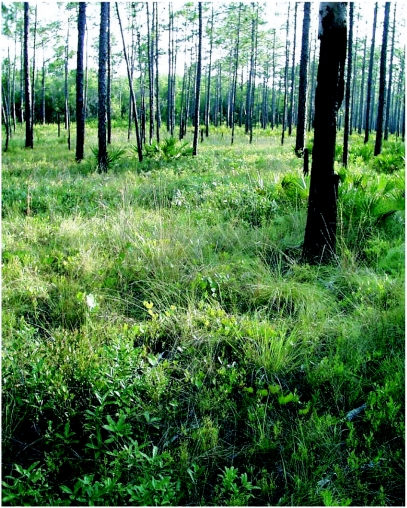
Longleaf pine (*Pinus palustris*) forest in the Apalachicola National Forest. *Polyergus lucidus* and its 3 most common hosts, *Formica archboldi*, *F*. *dolosa*, and *F. pallidefulva* found most commonly in this type of ecosystem in northern Florida.

Three colonies of *P*. *lucidus* were collected from pine flatwoods (Figure 1) of the Apalachicola National Forest in Leon County, Florida in June 2004 and February 2007. This forest occurs on flat topography, low elevation, and poorly drained, acidic, sandy soil (Abrahamson and Hartnett, 1990). It has an open overstory of pines (*Pinus palustris* Mill, and *P*. *elliottii* Engelm.) and a dense understory layer [the dominant species include Serenoa *repens* (W. Bartram) Small, *Ilex glabra* (L.) A. Gray, *Lyonia lucida* (Lam.) K. Koch, *Aristida beyrichiana* Trin. & Rupr., and other herbs] (Abrahamson & Hartnett, 1990). *Formica* colonies are most commonly found at the base of vegetation. Wire grass (*A. beyrichiana*) and runner oak (*Quercus pumila* Walt.) were the most common plants with which colonies were associated (Figure 1). Three queen right colonies each of *F*. *archboldi*, *F*. *dolosa*, and *F*. *pallidefulva* were also collected during this period.

Whole colony collections of all species were made by locating the central nest entrance and digging an approximately 0.5 m diameter cylinder of soil around the nest entrance to a depth of nearly 1 m. The soil was carefully sifted and all workers and brood were collected. All colonies collected were monogyne and monodomous. There was no evidence of satellite nests. Because nests of all three species were simple and relatively shallow, employing this method of nest excavation ensured entire collections for all colonies. Colonies were returned to the lab, censused, and cultured in large plastic trays lined with Fluon^™^. For observations, nests were established within a single, large plaster block (dental plaster, Castone^™^) with a clear glass cover and colonies were provided with water, sugar water (20% sucrose solution), and tenebrionid beetle larvae *ad libitum*. Laboratory colonies were maintained at 27–28 °C under constant light.

Additionally, laboratory observations were made on queen egg laying rates, nest organization, and interactions among workers, queens, and brood of both *P*. *lucidus* and *Formica* species. Behavioral repetoires for both host *F*. *dolosa* and *P*. *lucidus* *longicornis* were made from 12 hours of observations within the laboratory. An additional 8 hours of observation on in-nest behavior was made on the other two *Polyergus* colonies. Nest casts were made of colonies of *F*. *archboldi*, *F*. *dolosa*, and *F*. *pallidefulva* by W.R. Tschinkel. The methods and materials for nest casting are fully described by Tschinkel (2005a; b). Nest architecture was somewhat variable within species so one representative cast of a mature colony was selected to show differences among species. Colony frequency counts were made by walking four 10 m linear transects at each of the cardinal directions from each of the collected *Formica* colonies.

**Figure 2.**
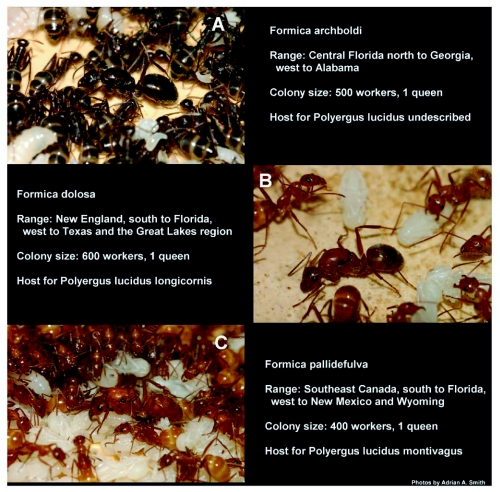
The 3 host *Formica* species for *P*. *lucidus* in the Apalachicola National Forest, their range, and average colony size in Florida. (A) *F*. *archboldi*, (B) *F*. *dolosa*, (C) *F*. *pallidefulva*.

## Results

### Formica

The endemic Nearctic *Formica pallidefulva* group of the genus *Formica* is a conspicuous group in pine flatwoods forests in northern Florida. Four of the 5 species in the *pallidefulva* group (*F*.*archboldi*, *F*. *dolosa*, *F*. *pallidefulva*, and *F*. sp. nov) occur in this region. Only the three known to host *P. lucidus* in this area: *F*. *archboldi*, *F*. *dolosa*, and *F*. *pallidefulva* (Figure 2) are discussed here. All three *Formica* species can typically be found at any given area in the Apalachicola National Forest. Nests of different species may be found as close as ∼ 2 m to one another, however, nests of the same species have never been found closer than ∼ 5 m to one another and are often much farther apart (JRK personal observation).

**Figure 3.**
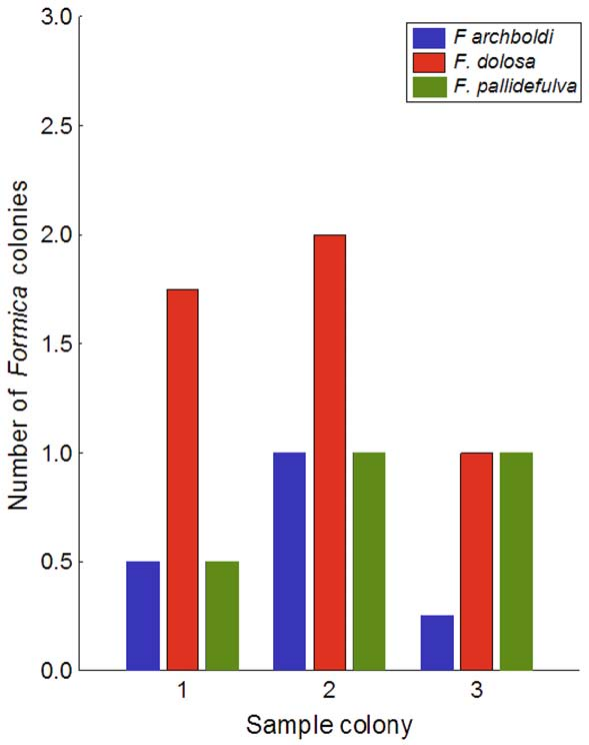
The average number of colonies surrounding any given *Formica* colony in the Apalachicola National Forest. Colonies were counted along 10 m transects at each of the four cardinal directions at each of 9*Formica* colonies (3 per species).

**Table 1.**
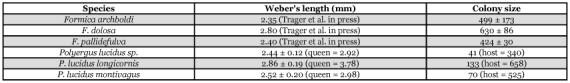
Body size (Weber's length in mm) and colony size for *Polyergus lucidus* and 3 host *Formica* species in Florida. Weber's lengths for *Formica* species are averages ± SD (JCT, unpublished data) while values for *Polyergus* are averages ± SD from this study. For *Polyergus* species, the Weber's length values in parentheses are for queens. Colony sizes for *Formica* are averages ± SD from this study (3 colonies each). Colony sizes for *Polyergus* are totals (1 colony) and include the number of *Polyergus* and *Formica* hosts (in parentheses).

Across northern Florida *F*. *dolosa* (Figure 2B) and *F. pallidefulva* (Figure 2C) were more abundant than *F*. *archboldi* (Figure 2A) and occur in a greater variety of upland ecosystems (JRK unpublished data). *Formica archboldi* was less common; restricted to pine flatwoods and sandhill in the region. *Formica dolosa* was the most abundant species among these species in the Apalachicola National Forest; locally often more than twice as common as *F*. *archboldi*, on average (Figure 3). *Formica dolosa* also had the largest average mature colony size (Table 1) and the largest worker body size (Table 1), although all species were slightly variable in size.

**Figure 4.**
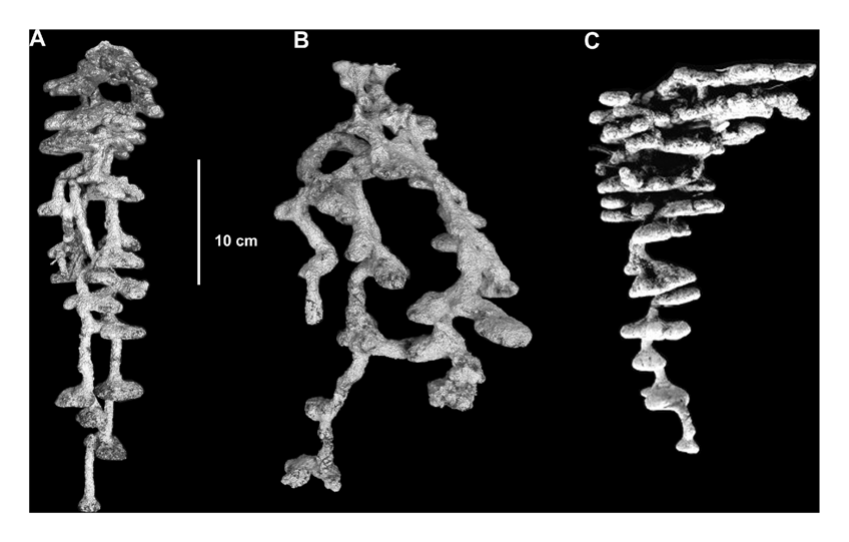
Nest architecture of (A) *F*. *archboldi*, (B) *F*. *dolosa*, and (C) *F*. *pallidefulva*

Nests of all *Formica* species were typically found at the base of vegetation. The nests were only visible from less than 3 m distance as they were often obscured by vegetation. The nests of *F*. *archboldi* were often the most difficult to locate because, in addition to their relative rarity, they were frequently at the base of wiregrass clumps that obscure excavated soil. The nest architecture of all three *Formica* species was variable within species, in the number of shafts, depth, and number of chambers (all of these features are also probably related to colony size: larger colonies tend to dig deeper nests, excavate a larger volume of soil, and have more shafts and chambers). There were, however, distinctive features common to each species. The nests of *F*. *dolosa* were the most obviously different from the other two species (Figure 4B). Specifically, there were often two or more nest entrances, the shafts tended to diverge from the central entrance, and the shafts tended to be much larger in diameter. The nests of *F*. *archboldi* (Figure 4A) and *F*. *pallidefulva* (Figure 4C) tended to have only one or two shafts that converged on the center of the vertical plane of their nests. Additionally, the shafts of their nests tended to be more obviously distinct, and smaller, than the chambers. Colonies were found to move after disturbance, including after raids by *Polyergus* colonies (Trager and Johnson 1985).

All of the *Formica* colonies collected for this study began to produce brood in March, even under laboratory conditions. Sexuals were produced by mature (non-incipient) colonies in the first round of brood. No mating flights were observed in the field. Under laboratory conditions, up to ten females and an equal or greater number of males were produced in the first round of brood. More sexual brood are produced by large, healthy colonies under natural conditions (JCT personal observation). One incipient colony of *F*. *dolosa* (8 minim workers) and one of *F*. *pallidefulva* (6 minim workers) were also collected in April 2007. These colonies produced only worker brood (like many temperate species, colonies do not produce sexuals in their first year). All of the colonies collected for this study were monogyne, although there are records of polygyny for *F*. *archboldi* elsewhere in Florida (Trager and Johnson 1985). Queens of *F*. *archboldi* (Figure 2A) and *F. pallidefulva* (Figure 2C) tend to be much larger than workers. However, *F*. *dolosa* queens are variable in size throughout their range. The *F*. *dolosa* queens collected for this study were only slightly larger than workers (Figure 2B).

Within laboratory nests, queens and brood were located centrally and attended by a circle of up to twenty workers (Figure 2). Under laboratory conditions the queens of all three species laid eggs at a rate of approximately 1 per hour. Younger workers, including callow workers, tended to be in close proximity to the queen and brood while older workers tended to be located at the periphery of the nest near the entrance or foraging, suggesting that a temporal worker caste system is operating for these species. Workers in queenless colonies of *F*. *dolosa* had functional ovaries and produced male offspring. Queenless colonies of *F*. *archboldi* and *F*. *pallidefulva* did not produce any brood in this study.

**Figure 5.**
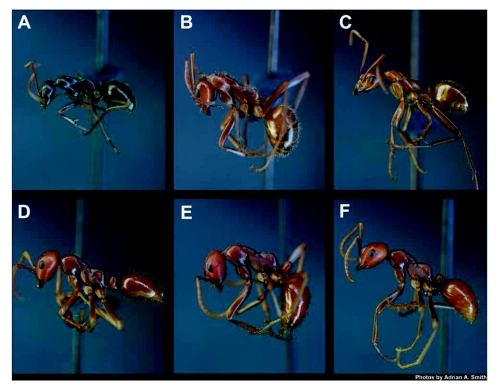
Workers of (A) *F*. *archboldi*, (B) *F. dolosa*, (C) *F*. *pallidefulva*, which are host to (D) *P. lucidus* un described, (E) *P*. *lutidus longicornis*, and (F) *P*. *lucidus montivagus*, respectively. The use of the subspecific variant names of *P*. *lucidus* should not be construed as a formal taxonomic entity.

Workers of all three species forage solitarily (Figure 5A-C), although they recruited up to several nestmates for larger food items or to honeydew-excreting hemipterans and nectaries that are close to the nest (Robson and Traniello 1998; JRK personal observation). These species were predaceous and were active scavengers as well, often found carrying dead insects back to their nest (Trager and Johnson 1985; JRK personal observation). All three species have also been observed actively tending membracids, aphids, and scales, most commonly on flowering palmetto (*S. repens*) and sapling pines (*P. palustris*) in the spring. *Formica archboldi* is probably a specialized predator and scavenger of the ponerine *Odontomachus brunneus* (Patton) (Trager and Johnson 1985), showing preference for this species over other ants and arthropods in field and laboratory trials (A.A. Smith and JRK, unpublished data). All species were diurnal foragers and were tolerant of high temperatures, often the only species found foraging at the height of the day (noon-3 pm) during the summer months (JRK personal observation). However the peak period of foraging was during morning (7 am-10 am) and evening (4 pm-6 pm) during the spring and summer. During the winter (∼ November – February) colonies (including foraging workers) are almost entirely inactive. A characteristic of both of these genera of formicines, including the species at the southern range limit in Florida, is that colonies overwintered in a relatively inactive state, without brood.

**Figure 6.**
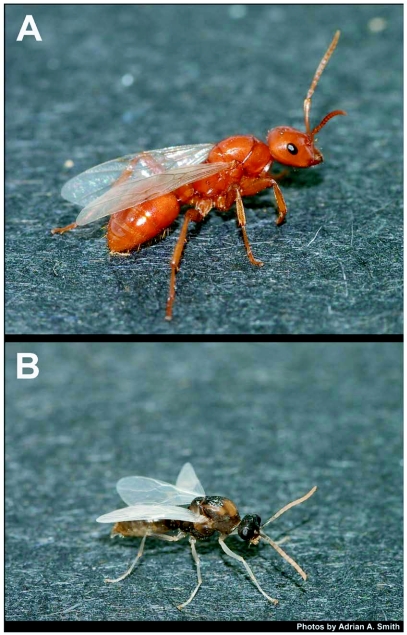
Alate queen (A) and male (B) of *P. lucidus undescribed*. The host of this species is *F*. *archboldi*.

### Polyergus

To avoid confusion and unnecessary repetition, throughout this section the subspecific names assigned to *P. lucidus* by Trager et al. in press are used. These names are useful because each subspecies is associated with enslaved hosts as follows: (1) *P. lucidus* undescribed with *F*. *archboldi*, *P. lucidus longicornis* with *F*. *dolosa*, and (3) *P*. *lucidus montivagus* with *F*. *pallidefulva* (Figures 2 and 5). The use of the subspecific variant names of *P*. *lucidus* should not be construed as a formal taxonomic change.

The *P. lucidus* undescribed colony and the *P. lucidus montivagus* colonies were collected with their hosts in February, 2006 less than 4 m from each other. Both of these colonies were collected in an overwintering state. That is, they were almost entirely inactive and devoid of brood. The *P*. *lucidus longicornis* colony was collected with its host in June, 2004 in a similar pine flatwoods habitat about 2 km east of the site where the other colonies were collected. Colonies and individuals of *P*. *lucidus* were similar in size to the average size of their respective hosts (Table 1). In all of these colonies, *P*. *lucidus* workers made up 12–20% of the number of workers (Table 1).

**Figure 7.**
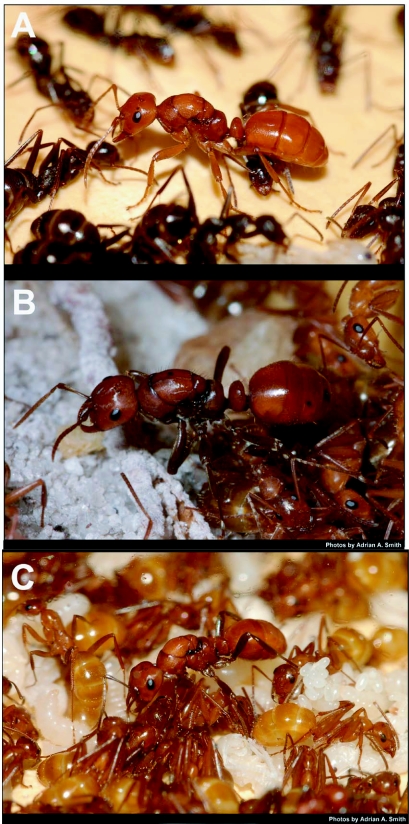
Queen, host workers, and brood of (A) *P*. *lutidus* undescribed with host *F*. *archboldi* and (B) *P*. *lucidus longicornis* with host *F*. *dolosa*, and (C) *P*. *lucidus montivagus* with host *F. pallidefulva*. Note that the imagesin A and B are at nearly the same scale, indicating a proportional size difference among the ants pictured, while C is reduced and thus not useful for casual estimation of size differences.

The *P*. *lucidus* undescribed and the *P*. *lucidus montivagus* colonies began to produce brood by March 2006, even under laboratory conditions. Sexuals were produced in the first round of brood by both colonies and consisted of 1 female and 7 males (*P*. *lucidus* undescribed, Figure 6) and 6 males (*P*. *lucidus montivagus*) that emerged as adults in May. The *P*. *lucidus longicornis* colony had 4 females and 18 males when it was collected in 2004 and 3 more females and 20 males emerged over the next month in the laboratory. No mating flights were observed. Queens of all 3 species were larger than the largest *Formica* workers in their respective colonies (Table 1, Figure 7) and *P*. *lucidus longicornis* was the largest queen, although the *P*. *lucidus* queens were all smaller than their *Formica* queen counterparts, except *P. lucidus longicornis* which was similar in size to the *F*. *dolosa* queens in the Apalachicola National Forest. This pattern was similar in the workers and *P. lucidus longicornis* (Figure 5E) was the largest of all the species (Table 1). The *P*. *lucidus* workers were mildly polymorphic and tended to be either similar in size or slightly larger than their *Formica* hosts (Table 1, Figure 5).

Like *Formica* colonies, within laboratory nests, queens and brood were located centrally and attended by a circle of up to twenty workers (Figure 7). Under laboratory conditions the queens of all three species laid eggs at a rate of approximately 1 per hour, however there were frequently gaps of up to 3 hours where eggs were not laid. Also like *Formica*, younger *Polyergus* workers, including callow workers, tend to be nearer to the queen and brood while older workers tend to be located at the periphery of the nest near the entrance or outside the nest, suggesting that a temporal worker caste system was operating for these colonies similar to that documented previously (Kwait and Topoff 1984). However, no *Polyergus* workers were ever within the circle of *Formica* workers that tend to the queen, nor did they spend more than a few seconds in her immediate vicinity.

**Table 2.**
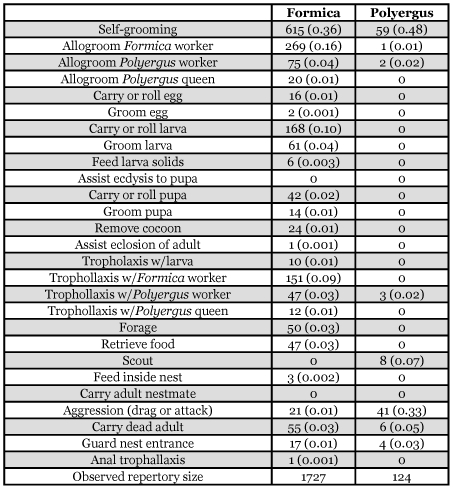
Ethogram of *Polyergus lucidus longicornis* and host *Formica dolosa* workers from the same colony. Observed frequencies are followed by values in parentheses indicating the frequency of each act relative to the total number of behaviors performed by the slaver and the host.

Queen dominance over *Polyergus* workers is obvious as they never interacted aggressively with her (Table 2). This is likely a chemical cue which may contribute to the spacing between *Polyergus* workers and the queen. In contrast, aggression in the form of biting, appendage pulling, and even occasionally spraying, is frequently observed between *Polyergus* workers and *Formica* workers, although the *Polyergus* workers are invariably the dominant individuals in the interspecies interactions (Table 2). Interestingly, *Formica* workers often acted aggressively toward their *Polyergus* queen. On two occasions (once with *P*. *lucidus longicornis* and once with *P*. *lucidus* undescribed), after several months in the laboratory the aggression of *Formica* workers escalated to the point that the queen was killed over the course of a few days of continuous harassment. This suggests that the chemical and behavioral cues that *Polyergus* queens employ to enslave their *Formica* hosts is, at best, imperfect and this probably contributes to the rarity of the species.

Observations of worker behavioral repertoire of the *P. lucidus longicornis* colony revealed that other than self grooming and raiding, *Polyergus* workers' only other tasks were occasionally removing dead of either species and trophallaxis with host *Formica* (Table 2). In contrast the behavioral repertoire of the host *Formica* workers consisted of dozens of tasks from nest maintenance, brood care, queen care, and foraging.

## Discussion

The rarity of *P*. *lucidus*, and slave-making species in general, limits our understanding of slave-making species. It is for this reason that we have presented as much natural history data on these species as possible, despite the paucity of colonies available. There is an absence of sociometric data (Tschinkel 1991) and in-nest behaviors for these species and their hosts in the literature (e.g. D'Ettorre and Heinze 2001). The implication of this shortcoming is that existing conceptual hypotheses about the biology and evolution of slave-making Formicinae lie upon a narrow ledge of empirical data. Most glaring is an absence of descriptive data such as colony size, body size, egg laying rates, and behavioral repertories of workers, for both the slave-making species and their hosts. Because these species are obligate parasites, the lack of information about the natural history of their hosts is most problematic (but see Savolainen and Deslippe 1996). These shortcomings could also impact conservation of these species as almost all of the slave-making ant species in the world are listed as threatened by the World Conservation Union (IUCN). Thus, the data presented here represent the best available information on *P. lucidus* until more colonies are found and catalogued.

For the most part, much of the natural history of *P. lucidus* and their hosts are similar, largely because the host workers perform most of the tasks necessary for colony function. It is for this reason that a description has been provided of all three host species and their natural history. In sum the collective natural history of these species indicate that *P*. *lucidus* is similar to and wholly dependent on their *Formica* hosts. The similarity in natural history is probably shaped by two factors: (1) the behavioral patterns of their host species are genetically based, and thus unchanged whether they are enslaved or not, and (2) because *Polyergus* are dependent on their host, their biology, such as brood production and ratio of workers in colonies (and colony size), is suited to exploiting their hosts. So, for example, colonies overwinter in a relatively inactive state and become active and begin producing brood in synchrony with their hosts. Similarly, colony size is probably closely matched to, or smaller than, their hosts because larger colonies may become unsustainable if local host colonies were wholly depleted of brood.

Among the five *Polyergus* species worldwide (*P*. *breviceps*, *P*. *lucidus*, *P*. *nigerrimus*, *P*. *rufescens*, *P. samurai*), *P*. *lucidus* subspecies and their *Formica pallidefulva* group hosts appear to have the smallest colony sizes. Here we report mixed colony sizes in the range of 400 – 600 workers while mixed colonies of *P*. *breviceps* / *F. podzolica*, *P*. *breviceps* / *F*. *gnava*, *P*. *rufescens* / *F*. *cunicularia*, and *P*. *samurai* / *F*. *japonica* are likely to be 3 to < 10 times larger (Kondoh 1968; Topoff et al. 1985; Savolainen and Deslippe 1996; D'Ettorre and Heinze 2001; Visicchio et al. 2003). Similarly, there is variability in the queen numbers, distribution, and ecology of all of the host species. The variability among these hosts and their slaves suggests that further study of the natural history of these species would be particularly valuable.

## Taxonomic implications of the co-occurrence of host races of *Polyergus lucidus*, *s.l*.

It is apparent from this study, the earlier one by Goodloe (1986) and unpublished data accumulated by JCT that the various nominate (and at least one unnamed) “subspecies” of *P*. *lucidus* have at least partially overlapping but distinct geographic ranges. The *Polyergus* taxa are uniquely associated with a particular *Formica pallidefulva*-*group* host species, and the geographic distribution of each is roughly coincident with the unique range of its respective host (Trager et al. in press). Preliminary revisionary studies by JCT indicate that the *P*. *lucidus* subspecies, including the undescribed one reported here and in Trager and Johnson (1985), have recognizable morphological attributes that together with their host - specificity and biogeography imply they would be better considered as full species. It is beyond the scope of this study to formalize these ideas, but we do suggest that until such time as a formal revision appears, it will be especially valuable for students of these ants to make every effort to properly identify the hosts according to the taxonomy of Trager, et al. in press. In the meantime we suggest that researchers also refer to the work of Smith (1947) for a more accurate rendition of the taxonomy than the overly simplified version presented by Creighton (1950) and especially, to collect, preserve and thoroughly label voucher specimens of coexisting host and parasite species from single nests (*Polyergus* home nest or nests they successfully raid).

## Conservation of a rare social insect

The genus *Polyergus* is one of the most curious, charismatic, and uncommon groups of ants in the world. The form of social parasitism that this genus practices also provides a model system for understanding one of the evolutionary quirks of eusocial behavior - dulosis - that is unique to the ants (D'Ettorre and Heinze 2001). The dependence of the *Polyergus* species on their host, their inherent rarity, and increasingly diminished available habitat suggest that they are likely candidates for local extinction throughout their range. For example, all three of the host species are sensitive to disturbance, particularly soil disturbance, and rarely, if ever, occur in highly disturbed areas such as urban landscapes, pastures, and roadsides in Florida (Trager and Johnson 1985; JRK unpublished data). However, *F*. *pallidefulva* is tolerant of disturbance in other parts of its range (Trager et al. in press).

*Polyergus lucidus* is a very rare insect in the Apalachicola National Forest. The three colonies described here were found over the course of 2 years of active searching across the (east to west) breadth of the Apalachicola National Forest and inspection of hundreds of *Formica* colonies by an expert collector (JRK) in this region. In the context of the abundance of their hosts, particularly *F*. *dolosa*, it is clear that *P*. *lucidus* is probably dependent on large populations of their hosts to persist. The taxonomic situation noted in the previous section (possibly three or more species rather than just one of *Polyergus* in eastern North America) lends still further import to the conservation of their populations and habitats. The longleaf pine forest in the southeastern U.S. contains some of the highest floral diversity of any temperate zone plant communities (Peet and Allard 1993). Longleaf pine now covers only 3% of its historical range and has become a threatened ecosystem. Although there has been considerable work on a few endangered vertebrate species in these ecosystems, particularly the red cockaded woodpecker (*Picoides borealis*), there is little known about the rare insect fauna. This study contributes to our knowledge of some of the rarest insects in this ecosystem. Because the Apalachicola National Forest is the largest remaining intact longleaf pine forest in the world and *Polyergus* seems to be more common and diverse here than elsewhere in its range, it is critical that this site remain protected and subject to appropriate ecological management.
